# Metagenomics dataset used to characterize microbiome in water and sediments of the lake Solenoe (Novosibirsk region, Russia)

**DOI:** 10.1016/j.dib.2020.106709

**Published:** 2020-12-30

**Authors:** Alla V. Bryanskaya, Aleksandra A. Shipova, Alexei S. Rozanov, Oxana A. Volkova, Elena V. Lazareva, Yulia E. Uvarova, Tatyana N. Goryachkovskaya, Sergey E. Peltek

**Affiliations:** a Laboratory of Molecular Biotechnologies of Federal Research Center Institute of Cytology and Genetics of the Siberian Branch of the Russian Academy of Sciences, 10 Lavrentiev Aven., Novosibirsk 630090, Russia; b Kurchatov Genomics Center of Federal Research Center Institute of Cytology and Genetics of the Siberian Branch of the Russian Academy of Sciences, 10 Lavrentiev Aven., Novosibirsk 630090, Russia; c The V.S. Sobolev Institute of Geology and Mineralogy SB RAS, 3 Koptyuga Aven., Novosibirsk 630090, Russia

**Keywords:** Metagenomics, Whole genome sequencing, Next-generation sequencing, Cyanobacterial community, Bottom sediments, Lake Solenoe

## Abstract

This is data on the microbial diversity in the floating cyanobacterial community and sediment samples from the lake Solenoe (Novosibirsk region, Russia) obtained by metagenomic methods. Such a detailed data of the microbial diversity of the Novosibirsk oblast lake ecosystem was carried out for the first time. The purpose of our work was to reveal microbial taxonomic diversity and abundance, metabolic pathways and new enzyme findings the studied lake ecosystem using the next-generation sequencing (NGS) technology and metagenomic analysis. The data was obtained using metagenomics DNA whole genome sequencing (WGS) on Illumina NextSeq and NovaSeq. The raw sequence data used for analysis is available in NCBI under the Sequence Read Archive (SRA) with the BioProjects and SRA accession numbers: PRJNA493912 (SRR7943696), PRJNA493952 (SRR7943839) and PRJNA661775 (SRR12601635, SRR12601634, SRR12601633) corresponding to floating cyanobacterial community and sediment layers samples, respectively.

## Specifications Table

**Table:** 

Subject	Microbiology: Microbiome
Specific subject area	Salt lake metagenomics, taxonomy and metabolic pathways
Type of data	Tables, Image, Figure and WGS sequencing data
How data were acquired	Paired-end sequencing of metagenomic DNA isolated from the floating cyanobacterial community and bottom sediment samples was performed using Illumina NextSeq platform, Illumina NovaSeq platform Bioinformatic analysis was carried out using the following programs: FastQC, Trimmomatic, CutAdapt, Bowtie2, MetaSPAdes, PhyloFlash and MG-Rast.
Data format	Raw and Analyzed
Parameters for data collection	Sampling; isolation of total DNA; library preparation; sequencing; bioinformatic processing and analysis. Total DNA was isolated from the floating cyanobacterial community and bottom sediment samples (a total of 15 specimens) using the Genomic DNA from soil NucleoSpin® Soil kit (Macherey-Nagel).
Description of data collection	The raw reads passed the quality control (with FastQC) and were trimmed using Trimmomatic and cutadapt. Contamination was reduced by mapping on the human genome using bowtie2. For taxonomic analysis, reads were compared to the SSU rRNA (SILVA) using phyloFlash. Metagenome assembly was performed using metaSPAdes. For automatic phylogenetic and functional analysis of the assembled metagenomes we used the MG-RAST server.
Data source location	Lake Solenoe (48), Novosibirsk region, Russia, 54°11′57.44″N 78°10′4.83″E
Data accessibility	Raw sequencing data Repository name: NCBI SRA. Data identification numbers: PRJNA493912 (SRR7943696), PRJNA493952 (SRR7943839) and PRJNA661775 (SRR12601635, SRR12601634, SRR12601633) Direct URL to data: https://trace.ncbi.nlm.nih.gov/Traces/sra/?run=SRR7943696, https://trace.ncbi.nlm.nih.gov/Traces/sra/?run=SRR7943839, https://trace.ncbi.nlm.nih.gov/Traces/sra/?run=SRR12601635, https://trace.ncbi.nlm.nih.gov/Traces/sra/?run=SRR12601634, https://trace.ncbi.nlm.nih.gov/Traces/sra/?run=SRR12601633; https://www.ncbi.nlm.nih.gov/bioproject/PRJNA493912, https://www.ncbi.nlm.nih.gov/bioproject/PRJNA493952, https://www.ncbi.nlm.nih.gov/bioproject/PRJNA661775 Metagenomics analysis of taxonomy and metabolic pathways Repository name: MG-RAST Data identification IDs: mgm4885516.3, mgm4885517.3, mgm4885518.3, mgm4885519.3, mgm4885520.3 Direct URL to data: https://www.mg-rast.org/linkin.cgi?project=mgp93351

## Value of the Data

• The obtained data can be used for investigation of microbial diversity and metabolic pathways and processes occurring in the unique hypersaline lake.
• Metagenomic data provide complete taxonomic profiles of microbial diversity and abundance in floating cyanobacterial community and sediment samples from the lake Solenoe (Novosibirsk region, Russia). The data may be very important for researchers which work in Bioinformatics, Biodiversity, Biochemistry, Biotechnology and other research areas.
• This is the first data on microbial diversity and metabolic pathways in cyanobacterial community and sediment samples obtained for the lake Solenoe. It can be used to compare taxonomic profiles and metabolic pathways of other ecosystems.

## 1 Data Description

The Solenoe lake belongs to the poorly studied lake system in the south of West Siberia. It is relatively small and weakly alkaline, with mineralization reaching 230 g/l in certain ears. In summer, dense or loose floating cyanobacterial communities form near the banks of the lake.

A description of the sampling points is given in Table 1.

**Table 1:** Description of the sampling points.

Station	Description
R1	Floating cyanobacterial community. Loose green mass concentrated in the upper layers of the water column.
R2	Loose layer of bottom sediments from the depth of 0–3 cm
R3	Bottom sediment from the depth of 3–5 cm
R4	Bottom sediment from the depth of 10–15 cm
R5	Bottom sediment from the depth of 15–19 cm

The raw sequencing data had the characteristics shown in Table 2.

**Table 2:** Sequence Read Archive (SRA) Data Description.

Sample name	Run	# of Spots	# of Bases
R1	SRR7943696	398,852,702	79.4G
R2	SRR7943839	398,845,300	79.4G
R3	SRR12601635	138,959,589	41.2G
R4	SRR12601634	21,981,321	6.5G
R5	SRR12601633	12,130,044	3.6G

Reads were extracted from each sample, aligned to the 16S rRNA gene sequence and identified to different taxonomic levels using the PhyloFlash program.

The metagenome of each sample were aligned on the bacterial and archaeal genome sequences and identified to different taxonomic levels using the MG-RAST program.

Taxonomic identifications of metagenomes of the samples obtained by 16S RNA and by WGS analyses are mostly similar despite some differences.

**Fig. 1:**
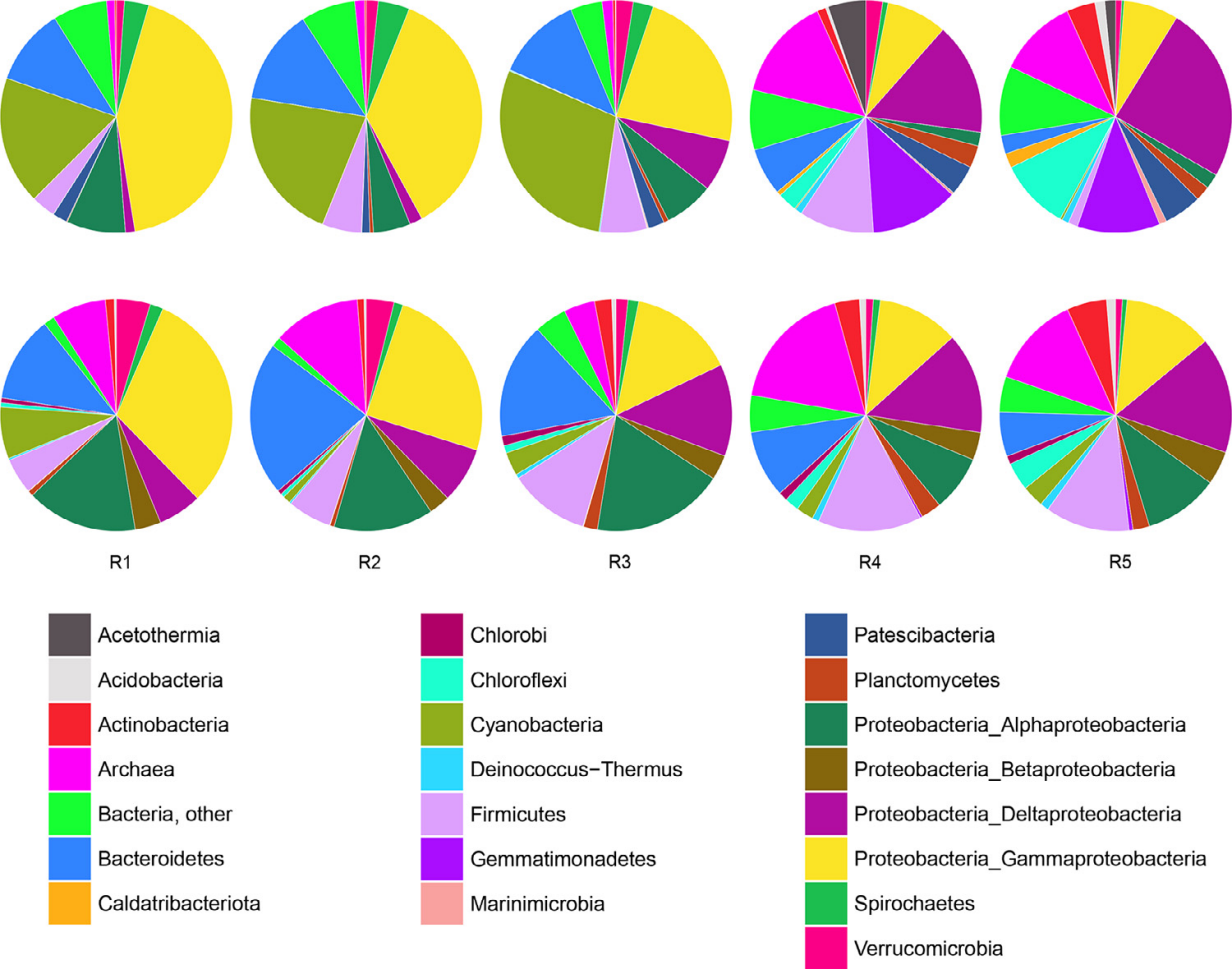
Taxonomic composition of the studied communities: Top line - Taxa according to WGS (16S RNA, PhyloFlash, SILVA) and Bottom line - Taxa according to WGS (metagenomes, MG-RAST, M5rna). R1 - cyanobacterial community; R2 – R5 bottom sediments.

The distribution of the phylums and classes of received OTUs (operational taxonomic units) is shown in Fig. 1.

In the studied samples, the percentage of Archaea was 1.1–14.4% according to PhyloFlash, and 4.4–17.9%, to MG-Rast; Euryarchaeota and Crenarchaeota were the most abundant archaeal phyla. The maximum percentage of Archaea was detected in the 4th layer of the bottom sediment (R4).

Among bacteria, Proteobacteria were the most abundant, especially in the upper layer (R1) where they accounted from 53.3% (PhyloFlash) and 56.6% (MG-Rast) of all microorganisms. Alphaproteobacteria were represented by up to 18.4% of the total sample; Deltaproteobacteria, by up to 24.7%; Gammaproteobacteria, up to 42.9%. Bacteroidetes were also abundant, especially in the R2 layer at 21.5% (MG-Rast). Cyanobacteria were the most frequent in R1, R2, and R3, with up to 29%. Firmicutes were found in all layers with the maximum of 14.4% in R4. Chloroflexi were the most abundant in the lowermost R5 layer at 9.9% (according to PhyloFlash).

Taxonomic composition of the studied communities: Top line, taxa according to WGS (16S RNA, PhyloFlash, SILVA); bottom line, according to WGS metagenomes

## 2 Experimental Design, Materials and Methods

### 2.1 Sample collection and description

The Solenoe lake is located near the village Lepokurovo (Bagan region, Novosibirsk oblast). The area of the studies was described by us earlier [1,2]. During our field studies (July 2017) the salinity of the lake was 122 g/l; рН, 8.46; Eh, 226 mV. Samples of the floating cyanobacterial community and bottom sediments were taken in the littoral zone in one point of the lake. First we sampled the mat, then pressed a plastic pipe 100 mm in diameter for sediment sampling. The samples were put into sterile 50 ml falcon tubes and kept in ethanol at −70 °C. Each sample was taken and analysed in triplicate.

In this study, the metagenome sample of the floating cyanobacterial community is referred to as R1; of the bottom sediments, as R2, R3, R4, and R5 (Table 1).

### 2.2 DNA extraction

Total DNA was isolated from the samples using the Genomic DNA from soil NucleoSpin® Soil kit (Macherey-Nagel) according to the manufacturer's protocol.

Total DNA extraction from deep bottom sediments was performed as follows: 300 µl of the sample was placed in a tube with ceramic beads for particle fragmentation and centrifuged for 1 min at 16 000 g. The supernatant was discarded, and 500 µl of the buffer containing 1000 mM Tris–HCl and 100 mM EDTA (pH 8.0) was added to the sediment. After vortexing, 100 µl of lysozyme (10 mg/ml) and 10 µl of RNAse were added and incubated at 37 °C for 25 min; every 5 min the tube was mixed. To this mixture, we added 100 µl each of 10% SDS (sodium dodecyl sulfate), 10% sarcosyl (Sigma), and chloroform. Samples were frozen in liquid nitrogen, then heated to 65°С and vortexed. This procedure was repeated three times, and then another three times after vortexing for 1 min and the addition of 100 µl of 10% polyvinylpyrrolidone (Sigma). The resulting mixture was centrifuged at 13 000 g for 10 min. The supernatant was transferred to a sterile tube with the equal volume of isopropanol and incubated for 30 min at - 20°С. The tube was centrifuged for 15 min at 16 000 g; 300 µl of 75% ethanol was added to the sediment, vortexed, centrifuged, and the supernatant was removed. This procedure was repeated one more time. After that, the sediment was dried at room temperature, dissolved in 150 µl of the buffer containing 10 mM Tris–HCl and 10 mM EDTA, and purified on CleanMag DNA magnetic particles (Eurogene) according to the standard protocol.

### 2.3 Sequencing and bioinformatic analysis

DNA nucleotide sequence libraries for sequencing (with average length 600 bp) were prepared using NEBNext (R) Ultra ™ DNA Library Prep Kit for Illumina ®. For the floating cyanobacterial community and the upper layer of bottom sediments, pair-end sequencing was performed by NovaSeq with NovaSeq 6000 S1 Reagent Kit (200 cycles) at the read length of 100 bp. For other bottom sediment layers, pair-end sequencing was performed by NextSeq with NextSeq 500/550 Mid Output Kit v2 (300 Cycles) at the read length of 150 bp. The quality of the reads was checked with the FastQC program. Then we used Trimmomatic (v. 0.36): with the CROP:97 MINLEN:95 options for the NovaSeq data [3]; with CROP:149 TRAILING:20 MINLEN:100, for the NextSeq data. Reads from the middle layer of bottom sediments were processed with cutadapt (v. 2.7) [4] with the options –nextseq-trim 20 -m 100. Contamination was reduced using bowtie2 (v. 2.3.5) by removing the sequences that could be mapped on the human genome [5]. Assembly of reads into contigs was done by metaSPAdes (v. 3.11.1), with the –only-assembler option, because the reads were already processed [6]. For the taxonomic analysis of microbial communities, we used the method implemented in phyloFlash v3.3b2 that is based on the alignment of reads against the SSU rRNA reference database [7]. This procedure was performed with the standard parameters. For automated phylogenetic and functional analysis of metagenomes we used the MG-RAST Web server v. 4.0.3 [8]. MG-RAST automatically does quality control, nucleotide and amino acid sequence alignments with database accessions. This allows one to perform taxonomic identification, detect metabolic pathways, and do comparative analysis of metagenomes. The data processing pipeline of MG-RAST includes read quality control (SolexaQA, DRISEE, Bowtie2) and annotation (FragGeneScan with the search against the protein M5nr database that integrates the GenBank, SEED, IMG, UniProt, KEGG, and eggNOGs databases).

## Ethics Statement

The work did not involve human subjects, animals, cell lines or endangered species of wild fauna and flora.

## CRediT Author Statement

**Alla V. Bryanskaya:** Conceptualization, Data curation, Writing. **Aleksandra A. Shipova:** Methodology, Software, Writing. **Alexei S. Rozanov:** Conceptualization, Data curation, Methodology. **Oxana A. Volkova:** Methodology, Software, Writing. **Elena V. Lazareva:** Conceptualization, Validation. **Yulia E. Uvarova:** Investigation. **Tatyana N. Goryachkovskaya:** Conceptualization, Validation. **Sergey E. Peltek:** Conceptualization, Validation, Supervision.

## Declaration of Competing Interest

The authors declare that they have not known competing financial interests or personal relationships which have, or could be perceived to have, influenced the work reported in this article.
